# 肺部切除术后慢性咳嗽预测模型的建立与验证

**DOI:** 10.3779/j.issn.1009-3419.2024.101.02

**Published:** 2024-01-20

**Authors:** Zhengwei CHEN, Gaoxiang WANG, Mingsheng WU, Yu WANG, Zekai ZHANG, Tianyang XIA, Mingran XIE

**Affiliations:** ^1^241001 芜湖，皖南医学院（陈郑玮）; ^1^Wannan Medical College, Wuhu 241001, China; ^2^230001 合肥，中国科学技术大学附属第一医院胸外科（王高祥，吴明胜，王宇，张泽锴，夏天洋，解明然 ）; ^2^Department of Thoracic Surgery, The First Affiliated Hospital of University of Science and Technology of China, Hefei 230001, China

**Keywords:** 肺部切除术, 术后慢性咳嗽, 中文版莱斯特咳嗽问卷, 预测模型, 列线图, 决策曲线分析, Pulmonary resection, Chronic cough, The Mandarin-Chinese version of Leicester cough questionnare, Prediction model, Nomogram, Decision curve analysis

## Abstract

**背景与目的** 肺部切除术后慢性咳嗽是最常见的并发症之一，严重影响患者术后生活质量，目前国内尚无关于肺部切除术后慢性咳嗽预测模型。因此，本研究旨在探讨肺部切除术后慢性咳嗽相关危险因素，构建预测模型并进行验证。 **方法** 回顾性分析2021年1月至2023年6月于中国科学技术大学附属第一医院接受肺部切除术的499例患者的临床资料和术后咳嗽情况，按7:3随机分配原则分为训练集（n=348）和验证集（n=151），根据训练集患者术后是否慢性咳嗽分为咳嗽组和非咳嗽组。使用中文版莱斯特咳嗽问卷（The Mandarin-Chinese version of Leicester cough questionnare, LCQ-MC）评估术前、术后咳嗽的严重程度及其对患者生活质量的影响，采用咳嗽视觉模拟量表（visual analog scale, VAS）和自拟的数字评分法（numerical rating scale, NRS）评估术后慢性咳嗽，采用单因素和多因素Logistic回归分析独立危险因素和模型构建，受试者工作特征（receiver operator characteristic, ROC）曲线评估模型区分度，校准曲线评估模型的一致性，绘制决策曲线分析（decision curve analysis, DCA）评估模型的临床应用价值。 **结果** 多因素Logistic分析筛选出术前用力呼气第1秒呼气量与用力肺活量比（forced expiratory volume in the first second/forced vital capacity, FEV_1_/FVC）、手术方式、行上纵隔淋巴结清扫、行隆突下淋巴结清扫、术后胸腔闭式引流时间是术后慢性咳嗽的独立危险因素，基于多因素分析结果构建列线图预测模型。ROC曲线下面积为0.954（95%CI: 0.930-0.978），最大约登指数所对应的临界值为0.171，此时敏感度为94.7%，特异度为86.6%。Bootstrap法抽样1000次，校准曲线图预测的肺部切除术后慢性咳嗽与实际发生风险高度一致。DCA显示当预测模型概率的预概率为0.1-0.9之间，患者表现为正的净收益。 **结论** 肺部切除术后慢性咳嗽严重影响患者生活质量。列线图的可视化展现形式有助于准确预测肺部切除术后慢性咳嗽，为临床决策提供支持。

## Abstract (en)

**Background and objective** Chronic cough after pulmonary resection is one of the most common complications, which seriously affects the quality of life of patients after surgery. Therefore, the aim of this study is to explore the risk factors of chronic cough after pulmonary resection and construct a prediction model. **Methods** The clinical data and postoperative cough of 499 patients who underwent pneumonectomy or pulmonary resection in The First Affiliated Hospital of University of Science and Technology of China from January 2021 to June 2023 were retrospectively analyzed. The patients were randomly divided into training set (n=348) and validation set (n=151) according to the principle of 7:3 randomization. According to whether the patients in the training set had chronic cough after surgery, they were divided into cough group and non-cough group. The Mandarin Chinese version of Leicester cough questionnare (LCQ-MC) was used to assess the severity of cough and its impact on patients' quality of life before and after surgery. The visual analog scale (VAS) and the self-designed numerical rating scale (NRS) were used to evaluate the postoperative chronic cough. Univariate and multivariate Logistic regression analysis were used to analyze the independent risk factors and construct a model. Receiver operator characteristic (ROC) curve was used to evaluate the discrimination of the model, and calibration curve was used to evaluate the consistency of the model. The clinical application value of the model was evaluated by decision curve analysis (DCA). **Results** Multivariate Logistic analysis screened out that preoperative forced expiratory volume in the first second/forced vital capacity (FEV_1_/FVC), surgical procedure, upper mediastinal lymph node dissection, subcarinal lymph node dissection, and postoperative closed thoracic drainage time were independent risk factors for postoperative chronic cough. Based on the results of multivariate analysis, a Nomogram prediction model was constructed. The area under the ROC curve was 0.954 (95%CI: 0.930-0.978), and the cut-off value corresponding to the maximum Youden index was 0.171, with a sensitivity of 94.7% and a specificity of 86.6%. With a Bootstrap sample of 1000 times, the predicted risk of chronic cough after pulmonary resection by the calibration curve was highly consistent with the actual risk. DCA showed that when the preprobability of the prediction model probability was between 0.1 and 0.9, patients showed a positive net benefit. **Conclusion** Chronic cough after pulmonary resection seriously affects the quality of life of patients. The visual presentation form of the Nomogram is helpful to accurately predict chronic cough after pulmonary resection and provide support for clinical decision-making.

肺癌的死亡率在全世界范围内一直居所有恶性肿瘤之首^[1⇓-3]^。近年来，随着低剂量螺旋计算机断层扫描（low dose spiral computed tomography, LDCT）的广泛应用及定期体检的普及，越来越多的肺结节患者被检出，其中部分患者需要接受手术治疗。咳嗽是肺部手术后最常见的并发症之一，术后急性咳嗽控制不佳将转变为慢性咳嗽。文献^[4⇓⇓-7]^报道，肺部手术后慢性咳嗽发生率在25%-50%之间。慢性咳嗽会加剧患者术后切口的疼痛，使其怀疑治疗效果，甚至会导致部分患者出现抑郁症状，严重影响术后生活质量^[8]^。因此，研究肺部手术后慢性咳嗽发生规律，并在围手术期给予适当干预，减少其发生率并改善患者症状，是临床亟待解决的问题之一。目前，国内外文献对肺部手术后慢性咳嗽危险因素相关性分析较少，未有报道关于肺部手术后慢性咳嗽模型建立。因此，本研究旨在构建肺部术后慢性咳嗽的临床预测模型，以期为肺部手术患者围手术期预防和管理提供支持，减少肺部术后慢性咳嗽的发生率。

## 1 资料与方法

### 1.1 研究对象

回顾性分析2021年1月至2023年6月于中国科学技术大学附属第一医院接受单孔胸腔镜肺部切除术的499例患者临床资料和术后咳嗽情况，按7:3随机分配原则分为训练集（n=348）和验证集（n=151）。纳入标准：（1）接受单孔胸腔镜肺叶切除术或亚肺叶切除术；（2）组织病理学证实为周围型非小细胞肺癌；（3）R0切除；（4）无新辅助治疗；（5）签署知情同意书。排除标准：（1）术前存在呼吸道感染性疾病，如咽炎、过敏性鼻炎、慢性阻塞性肺疾病（chronic obstructive pulmonary disease, COPD）、支气管哮喘、鼻后滴流综合征等；（2）手术中转开放手术；（3）中央型肺癌或接受袖式切除或气管重建手术；（4）术后出现严重并发症，包括肺部感染、明显神经损伤、肺栓塞、乳糜胸等；（5）失访或病例资料不完整。本研究经中国科学技术大学附属第一医院伦理审查委员会批准（2023-RE-379）。

### 1.2 研究方法

收集受试者性别、年龄、吸烟史、高血压史、糖尿病史、术前肺功能指标、肿瘤部位、肿瘤最大径、手术麻醉时间、术中是否双腔管插管、胸腔是否粘连、手术左右侧、手术方式、是否位于上叶、淋巴结清扫方式、上纵隔淋巴结清扫情况、下纵隔淋巴结清扫情况、术后胸引管带管时间。其中淋巴结清扫方式主要包括选择性淋巴结采样和系统性淋巴结清扫。选择性淋巴结采样对手术中可疑转移的淋巴结进行采样活检；系统性淋巴结清扫指系统性清除解剖标志内包含淋巴结在内的所有纵隔组织，要求最少切除3站纵隔淋巴结，并且其中必须包括隆突下淋巴结，除纵隔淋巴结以外，肺门和肺内淋巴结必须一并切除^[9]^。

通过电话随访调查问卷的方式比较两组患者肺术后慢性咳嗽情况及生活质量情况。本研究使用中文版本的莱斯特咳嗽量表^[10]^（Mandarin-Chinese version of the Leicester cough questionnaire, LCQ-MC）评估患者咳嗽严重程度及对生活质量的影响，LCQ-MC分为生理、心理和社会3个维度，一共19道题，包括8项生理项目、7项心理项目和4项社会项目，每道题7个选项（正向计分，1-7个等级，分数越高表示咳嗽程度越轻）。各维度的得分由各维度题目分值取平均值（1-7分），总分为3个维度得分之和（3-21分）。本研究中的499例患者均在2位经过培训的医务人员的指导下，分别于手术前1天、术后8周完成LCQ-MC。根据训练集患者术后8周是否合并慢性咳嗽将其分为发生咳嗽组（n=94）及非咳嗽组（n=254）。

### 1.3 咳嗽评价方法

术后慢性咳嗽采用咳嗽视觉模拟量表（visual analog scale, VAS）和自拟的数字评分法（numerical rating scale, NRS）共同评估。VAS是一种线性评分方法，使用0-100的刻度线，0表示没有咳嗽，100表示最严重的咳嗽，要求患者根据自己对咳嗽的知觉在刻度线上标记咳嗽的严重程度，并且以测量起始点到标记点的距离作为评分，分数越高，咳嗽越严重。NRS评价患者的咳嗽程度（0分：无咳嗽；1-3分：轻度咳嗽，对睡眠无影响；4-6分：中度咳嗽，对睡眠有影响，但能入睡；7-9分：重度咳嗽，无法入睡或睡眠中咳醒；10分：剧烈咳嗽）。当VAS刻度线达到60 mm且NRS评分达到4分时，可将该患者纳入术后慢性咳嗽组；当VAS评分或NRS评分只满足一项或者两项都未达到时纳入非咳嗽组。

### 1.4 统计学分析

应用SPSS 26.0进行数据分析，P<0.05为差异有统计学意义。应用卡方检验、t检验与秩和检验比较两组患者临床病例资料，应用单因素二元Logistic回归分析评估肺部手术后慢性咳嗽的相关性，仅对训练集中单因素分析有统计学意义的因素，进一步纳入多因素二元Logistic回归分析，以寻找独立危险因素（包括临床因素、术中因素、术后因素）。将最终独立危险因素引入R软件（The R Foundation for Statistical Computing, Vienna, Austria）4.3.2版，采用“pROC”进行受试者工作特征（receiver operator characteristic, ROC）曲线分析。Nomogram用“rms”完成，决策曲线分析（decision curve analysis, DCA）用“rmda”完成。曲线下面积（area under the curve, AUC）均采用自举偏差校正的95%CI。同时建立ROC，采用AUC评估此模型；为了评估Nomogram的临床应用价值，使用临床校准曲线展示预测数据和实际数据之间拟合情况；通过计算一系列阈值概率的净收益，将数据集进行DCA分析。

## 2 结果

### 2.1 两组患者单因素分析

将499例患者按7:3随机分配原则分为训练集（n=348）和验证集（n=151），其中训练集中咳嗽组94例，非咳嗽组254例；男性139例，女性209例。通过单因素分析结果发现两组患者性别、吸烟史、高血压史、糖尿病史、是否位于上叶、手术左右侧的差异均无统计学意义（P>0.05），两组患者年龄、是否双腔管插管、胸腔是否粘连、手术方式、淋巴结清扫方式、是否行上纵隔淋巴结清扫、是否行隆突下淋巴结清扫、肿瘤最大径、FEV_1_、FVC、FEV_1_/FVC、MVV、麻醉时间、术后带管时间的差异均有统计学意义（P<0.05），见表1。

**表 1:** 咳嗽组与非咳嗽组单因素分析结果（训练集）

Variables	Cough group (n=94)	Non-cough group (n=254)	χ^2^/Z/t	P
Gender			0.366	0.545
Male	40 (42.6%)	99 (39.0%)		
Famale	54 (57.4%)	155 (61.0%)		
Age (yr)			11.025	0.001
<60	45 (47.9%)	171 (67.3%)		
≥60	49 (52.1%)	83 (32.7%)		
Smoking			0.593	0.441
Yes	9 (9.6%)	18 (7.1%)		
No	85 (90.4%)	236 (92.9%)		
Hypertension			0.058	0.810
Yes	26 (27.7%)	67 (26.4%)		
No	68 (72.3%)	187 (73.6%)		
Diabetes			0.687	0.407
Yes	7 (7.4%)	13 (5.1%)		
No	87 (92.6%)	241 (94.9%)		
Double-lumen tubes			9.336	0.002
Yes	93 (98.9%)	225 (88.6%)		
No	1 (1.1%)	29 (11.4%)		
Adhesion			7.264	0.007
Yes	23 (24.5%)	32 (12.6%)		
No	71 (75.5%)	222 (87.4%)		
Side of operation			0.001	0.982
Right	64 (68.1%)	150 (59.1%)		
Left	30 (31.9%)	104 (40.9%)		
Method of operation			119.718	<0.0001
Wedge-shape	8 (8.5%)	104 (40.9%)		
Segmentectomy	19 (20.2%)	119 (46.9%)		
Lobectomy	67 (71.3%)	31 (12.2%)		
Upper lobe			1.571	0.210
Yes	66 (70.2%)	160 (63.0%)		
No	28 (29.8%)	94 (37.0%)		
Lymphadenectomy			25.398	<0.0001
Sample or non-sample	19 (20.2%)	16 (6.3%)		
Systematic cleaning	75 (79.8%)	238 (93.7%)		
Superior mediastinal lymph			206.173	<0.0001
Yes	75 (79.8%)	12 (4.7%)		
No	19 (20.2%)	242 (95.3%)		
Lower mediastinal lymph			103.393	<0.0001
Yes	81 (86.2%)	65 (25.6%)		
No	13 (13.8%)	189 (74.4%)		
Maximum diameter^a^	18 (12, 25)	9 (7, 12)	-8.940	<0.0001
FEV_1_	2.38±0.62	2.60±0.65	-2.937	0.004
FVC	3.21±0.79	3.44±0.77	-2.385	0.018
FEV_1_/FVC (%)^a^	74.5 (69, 79)	77 (72, 82)	-2,901	0.002
MVV	86.19±20.11	90.96±19.61	-1.999	0.046
Anesthesia duration^a^	150 (120, 185)	120 (90, 150)	-5.718	<0.0001
Duration of drainage tube^a^	4 (3, 6)	3 (2, 3)	-7.262	<0.0001

^a^Data is represented by median (P_25_, P_75_). FEV_1_: forced expiratory volume in the first second; FVC: forced vital capacity; MVV: maximal voluntary ventilation.

### 2.2 两组患者多因素分析

将单因素分析有意义的结果纳入多因素分析显示：FEV_1_/FVC（P=0.047）、手术方式（P=0.044）、行上纵隔淋巴结清扫（P<0.001）、行隆突下淋巴结清扫（P=0.004）、术后带管时间（P=0.005）是单孔胸腔镜肺切除术后慢性咳嗽组与非咳嗽组的独立危险因素，见表2。

**表 2:** 咳嗽组与非咳嗽组多因素分析结果（训练集）

Variables	B	Calibration error	P	Exp(B)	OR (95%CI)
Age	0.886	0.531	0.095	2.426	0.856-6.876
FEV_1_	3.373	1.746	0.053	29.154	0.951-893.863
FVC	-1.528	1.373	0.266	0.217	0.015-3.198
FEV_1_/FVC (%)	-0.128	0.064	0.047	0.880	0.775-0.998
MVV	-0.049	0.026	0.058	0.952	0.905-1.002
Anesthesia duration	0.006	0.004	0.145	1.006	0.998-1.015
Double-lumen tubes	-0.856	1.308	0.513	0.425	0.033-5.516
Maximum diameter	0.045	0.029	0.123	1.046	0.988-1.109
Lymphadenectomy	-0.179	1.039	0.863	0.836	0.109-6.410
Surgical procedure			0.044		
Segmentectomy vs Wedge-shape	-0.965	0.697	0.166	0.381	0.097-1.492
Lobectomy vs Wedge-shape	-0.529	0.607	0.383	0.589	0.179-1.936
Superior mediastinal lymph	-4.063	1.070	<0.001	0.017	0.002-0.140
Lower mediastinal lymph	-1.462	0.504	0.004	0.232	0.086-0.622
Duration of drainage tube	0.213	0.075	0.005	1.237	1.067-1.434

OR: odds ratio; CI: confidence interval.

### 2.3 术前、术后8周LCQ-MC评分比较

所有患者术后随访8周，咳嗽组与非咳嗽组术前LCQ-MC评分无统计学差异（19.96±0.44 vs 19.70±0.27, P=0.261），术后8周LCQ-MC评分咳嗽组明显低于非咳嗽组，差异有统计学意义（15.82±1.01 vs 19.18±0.34, P<0.001），术前及术后LCQ-MC克朗巴赫α系数分别为0.671及0.961。

### 2.4 列线图模型构建

根据多因素分析中P<0.05的结果构建肺部手术后慢性咳嗽预测模型列线图。最终将FEV_1_/FVC（P=0.047）、手术方式（P=0.044）、行上纵隔淋巴结清扫（P<0.001）、行隆突下淋巴结清扫（P=0.004）、术后带管时间（P=0.005）等5个独立危险因素纳入列线图的构建（图1）。根据列线图模型中各个风险因素对结局变量的影响程度，对每个变量不同水平进行打分（行肺段切除：15分，肺叶切除：32.5分，上纵隔淋巴结清扫：100分，隆突下淋巴结清扫：45分，FEV_1_/FVC与术后带管时间对应得分），然后将各个变量得分相加得到总分；最后通过总评分计算结局事件发生概率，从而计算该结果的预测值大小。

**图 1:**
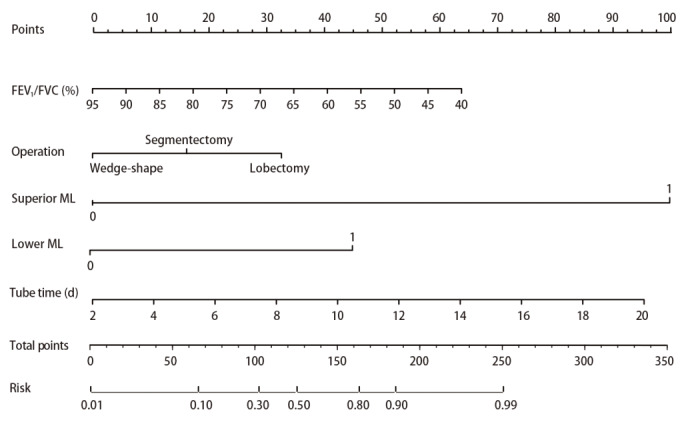
肺部术后慢性咳嗽列线图构建

### 2.5 预测模型验证与效能评价

预测模型的区分度：将多因素分析后结果带入该模型，预测肺部手术后慢性咳嗽的ROC AUC为0.954（95%CI: 0.930-0.978），最大约登指数所对应的临界值为0.171，此时敏感度为94.7%，特异度为86.6%；外部验证集ROC AUC为0.897（95%CI: 0.842-0.952），最大约登指数所对应的临界值为0.861，此时敏感度为85.5%，特异度为90.2%，提示本预测模型具有较好的区分度（图2）。

**图 2:**
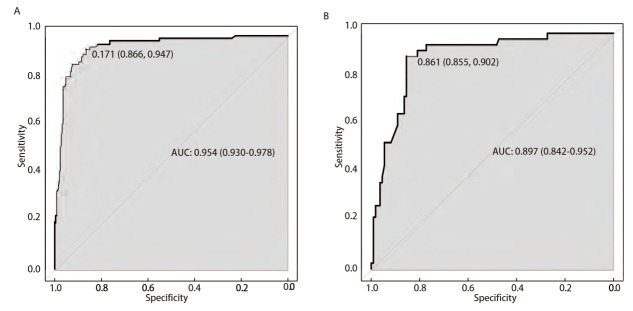
肺部术后慢性咳嗽ROC曲线构建。 A：训练集；B：验证集。

预测模型的校准度：通过加强Bootstrap法重复抽样1000次进行内外部验证，校准曲线显示的预测值与实际值高度吻合（图3），训练集平均绝对误差为0.026，验证集平均绝对误差为0.013，提示使用校准曲线发现预测数据和实际数据之间有显著的联系，校准曲线图预测的肺部手术后慢性咳嗽预测风险与实际发生风险高度一致。

**图 3:**
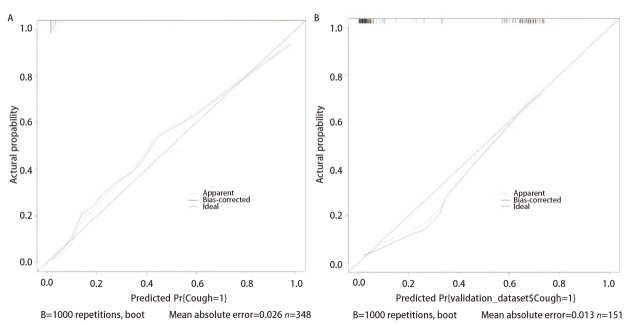
肺部手术后慢性咳嗽校准曲线分析。 A：训练集；B：验证集。

预测模型的适用度：DCA显示模型的阈概率为0.1-0.9（图4），模型表现为正的净收益，图中两条曲线代表两种极端情况，标“无”的横线表示所有患者均为接受肺切除术，且不进行干预，净收益为0；标“全部”的斜线表示所有患者均接受肺切除术，并实施干预所获得的净收益。红色的曲线是采用列线图预测模型下患者所获得的临床净收益。

**图 4:**
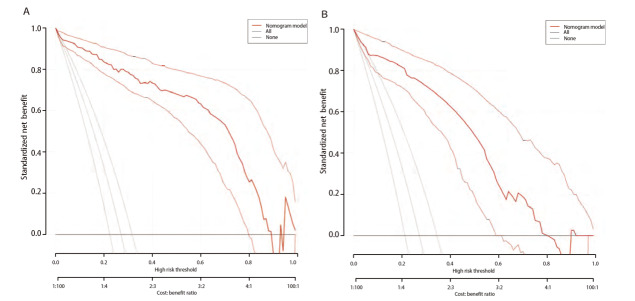
肺部手术后慢性咳嗽临床DCA。 A：训练集；B：验证集。

## 3 讨论

肺部手术后咳嗽是最常见且难治的并发症之一，控制不佳将转变为慢性咳嗽^[11]^。美国胸科学会对慢性咳嗽定义为：咳嗽为唯一或者首要症状且持续时间超过8周，并且无肺部疾病的影像学证据^[12]^。术后慢性咳嗽症状会增加患者焦虑情绪，不可避免地产生心理和生理创伤，严重影响患者术后的生活质量。术后慢性咳嗽受到临床内外持续关注，对于引发肺术后慢性咳嗽的危险因素进行探索并在术中超前干预及采取合理防治措施是患者良好预后的关键；术后慢性咳嗽可能与手术因素^[13]^、肺容量损失继发病变^[14]^、麻醉因素^[15]^和其他因素相关。本研究通过构建肺部手术后慢性咳嗽模型，为术中临床决策及术后管理提供支持，有效减少肺部手术后慢性咳嗽发生，提高患者术后生活质量。

肺部手术后慢性咳嗽的机制目前研究较少，且存在一定争议。既往研究^[16]^表明纵隔淋巴结清扫是肺部手术后慢性咳嗽的重要危险因素，特别是上纵隔淋巴结清扫与隆突下淋巴结清扫对肺部手术后慢性咳嗽的影响；我们研究发现行上纵隔淋巴结清扫与隆突下淋巴结清扫肺部手术后慢性咳嗽的发生率更高。Mu等^[17]^回顾性分析901例肺切除患者的术后持续咳嗽的特征，结果显示纵隔淋巴结清扫是肺部手术后持续性咳嗽的独立危险因素。对于上纵隔淋巴结清扫与隆突下淋巴结清扫可能加重术后慢性咳嗽的机制，我们分析可能与以下原因相关：（1）术中在清扫隆突下淋巴结会留下一个残腔，导致肺部快速适应性牵张感受器暴露在外，术后患者下床活动引起的机械性刺激和胸腔积液引起的化学性刺激就会兴奋这些感受器，通过迷走神经的传递引起咳嗽反射，从而引起术后咳嗽的发生；（2）肺术后持续性咳嗽很大程度上是由于神经C纤维的刺激引起的^[18]^，而肺部咳嗽的70%-80%的感觉神经纤维是由神经C纤维构成的，术中电刀、超声刀等能量器械进行操作时会损伤位于相应位置迷走神经传入纤维和气管壁导致咳嗽传入纤维敏感性增加和气管壁内的咳嗽感受器异常兴奋从而加重术后咳嗽；（3）隆突下淋巴结的清扫会引起周围气道和相邻肺组织发生炎症反应，通过多种途径释放如缓激肽（bradykinin, BK）和前列腺素E2（prostaglandin E2, PGE2）等炎症因子，在此基础上激活瞬时受体电位通道香草醛亚型1（transient receptor potential channel vanillin subtype 1, TRPV1）通路^[19]^，从而通过不同的途径诱发术后咳嗽。另外，不同手术方式同样影响着肺部手术后慢性咳嗽的发生率，Chen等^[6]^研究表明，行双腔导管麻醉下肺大疱切除术3个月后，咳嗽的概率约为50%，肺段切除术和肺叶切除术约为60%，由此可见肺段及肺叶切除术较楔形切除术更容易发生术后咳嗽，我们的研究结果与此类似，我们分析原因为：（1）这与离断患者的断支气管角度过长与角度扭曲有关，会激活肺组织内巨噬细胞产生炎症反应，从而诱发咳嗽的发生；（2）肺段切除及肺叶切除更容易损伤段支气管旁局部神经纤维，导致咳嗽相关神经元通路受损，引起术后慢性咳嗽。

近年来，研究^[20,21]^发现术前肺功能训练可以预防肺部手术后咳嗽的发生。Hasanpour等^[20]^通过对81例COPD患者进行多模式的肺部呼吸功能训练，结果显示训练组3个月后FEV_1_/FVC有明显的改善，显著减少COPD患者的咳嗽喘息方面的症状。我们研究发现患者肺功能差、手术前FEV_1_/FVC越低，术后慢性咳嗽发生率越高。我们分析可能原因有：（1）咳嗽作为人体的一种保护性呼吸反射动作，FEV_1_/FVC越低，提示小气道通气功能越差，当肺部手术后肺容积缩小时，会主动咳嗽，促进肺复张。（2）肺功能较差患者呼吸道防御保护作用下降，对于肺部手术后残端更容易形成局部炎症，炎症过程酸性物质的积累和气道pH值的变化可刺激相应位置的咳嗽感受器，通过一系列神经传递引起咳嗽反射的发生，从而加重术后咳嗽。目前，关于胸腔闭式引流时间对肺部术后慢性咳嗽研究较少；我们研究发现术后胸腔闭式引流时间越长，患者术后慢性咳嗽发生率越高，生活质量越低。Huang等^[21]^通过观察行肺癌根治+纵隔淋巴结切除术的100例患者，发现脂肪填充组在拔出胸引管4周后，患者的夜间咳嗽明显改善。我们分析原因可能是：（1）胸腔闭式引流管对胸膜的刺激，可导致患者呼吸肌肌力下降、肺容量减少，使得分泌物滞留呼吸道，导致术后慢性咳嗽的发生；（2）引流管留置时间延长及术后切口疼痛感愈强通常限制术后早期的身体活动，胸管可以促进胸腔积液的引流和胸腔减压，无论是机制牵引还是胸腔积液都不会过度刺激咳嗽受体；当置管时间延长，胸腔积液减少，负压增加时反而刺激咳嗽受体，导致咳嗽的发生。

列线图是一种用于临床事件个体化预测分析的统计学模型，与其他预测性统计学方法相比，列线图分析可以通过直观且可视化的方式提供更好的个体化预后风险评估。本研究将多因素Logistic回归分析中的独立危险因素对肺部术后慢性咳嗽影响的权重绘制Nomogram模型，对每个变量不同水平进行打分，总分越高的患者肺部术后发生慢性咳嗽的风险越大。经验证显示模型区分度良好，其预测值与观察值校准度良好，同时患者的临床获益可观。临床工作中根据此模型可以精准地预测肺部术后慢性咳嗽的发生，术前肺功能训练、术中超前干预及术后合理防治将有效减少术后慢性咳嗽的发生。

综上所述，基于患者的术前FEV_1_/FVC、手术方式、行上纵隔淋巴结清扫、行隆突下淋巴结清扫、术后胸腔闭式引流时间制作的肺部术后慢性咳嗽列线图精准地预测了术后慢性咳嗽的发生，术前、术中和术后有针对性的干预是减少术后慢性咳嗽的有效途径，列线图预测模型是减少术后慢性咳嗽的有效工具。由于本研究是单中心回顾性研究，这些结果可能会受到选择偏倚的影响，进一步的研究应该为前瞻性且多中心入组更多的患者，以便更好地验证肺部切除术后慢性咳嗽预测模型。
